# Athlete Leadership Development Within Teams: Current Understanding and Future Directions

**DOI:** 10.3389/fpsyg.2022.820745

**Published:** 2022-02-17

**Authors:** Stewart T. Cotterill, Todd M. Loughead, Katrien Fransen

**Affiliations:** ^1^School of Rehabilitation, Sport and Psychology, AECC University College, Bournemouth, United Kingdom; ^2^Department of Kinesiology, University of Windsor, Windsor, ON, Canada; ^3^Department of Movement Sciences, KU Leuven, Leuven, Belgium

**Keywords:** leadership development, mentoring, peer leadership, shared leadership, athlete leadership

## Abstract

Leadership has been shown to be a fundamental factor influencing the performance of sport teams. Within these teams, leadership can be provided by coaches, formal athlete leaders, such as team captains, and other ‘informal’ athlete leaders. The role of the athlete leader in a team, either formal or informal, has been consistently reported over the last 10 years to have a significant impact upon a teams’ functioning and effectiveness, as well as teammates’ general health and mental wellbeing. As such, cultivating the provision of this leadership within a team has emerged as an important focus for managers, coaches, sport psychologists and scholars alike. While the recognition of the importance of athlete leadership is well established, there has been a lag in the development of systematic approaches to enhance and develop the leadership skills and capabilities of the athletes within sport teams. As a result, this paper seeks to review contemporary examples and current understanding of approaches to athlete leadership development. The paper will also highlight future areas for research and applied practice development.

## Introduction

Leadership is a fundamental aspect of sport, particularly as it relates to the effectiveness of teams within sport environments (Cotterill and Fransen, 2016). The concept of leadership has been examined across a wide range of contexts, both within and outside of sport, which has led to a broad spectrum of leadership definitions and theories. However, the common features of these various conceptualisations of leadership are that leadership is a process that involves influencing others, occurs within the context of a group and focuses on the attainment of common goals (Northouse, 2018). In current conceptualisations of leadership in organisational settings, team leadership has been recognised as a distinct form of organisational leadership (Kozlowski et al., 2016). That is, team leadership can be viewed as any individual fulfilling a team’s needs. Within sport teams, team leadership can stem from coaches, formal athlete leaders, such as team captains, but also informal athlete leaders. This leadership of and within sport teams has emerged as an important focus for managers, coaches, sport psychologists and scholars alike (Day et al., 2014). Athlete leadership has been defined more specifically as ‘an athlete, occupying a formal or informal role within a team, who influences a group of team members to achieve a common goal’ (Loughead et al., 2006, p. 144). Athlete leaders have been reported to positively influence team cohesion, athlete satisfaction, team identification, team confidence and the motivational climate within the team (Cotterill and Fransen, 2016). Recent work has reported that the leadership needs of a sport team exceed the capabilities of one individual, and it is often multiple persons in the team who occupy the different leadership roles on and off the field (e.g., Fransen et al., 2014; Duguay et al., 2019). The leadership needs within a sports team can be met in a range of different ways by different individuals undertaking different roles including, coaches, team captains and informal athlete leaders (Mertens et al., 2021).

However, while there has been an increasing focus on understanding leadership within sports teams, and athlete leadership in particular in recent years (Cotterill and Fransen, 2016), far less attention has been paid to approaches to the development of leadership within teams. As a result, the aim of this review is to clarify current understanding regarding the sources of leadership within sports teams, and then crucially to review current understanding regarding the development of leadership and leaders within sports teams.

### The Different Leadership Sources in Sport Teams

#### Team Captains

In many sport teams, the captain (i.e., the formal athlete leader in the team) is perceived to fulfil an important leadership function. Indeed, it has been suggested that good captaincy can have a marked impact upon performance (Cotterill and Fransen, 2016). The captaincy role itself is something that has historically suffered from a lack of clarity. Several different roles and responsibilities for team captains have been suggested over the past 50 years. For example, Mosher (1979) outlined three main responsibilities, which are to act as a liaison between the coaching staff and the team, to be a leader during all team activities and to represent the team at events, meetings and press conferences. In addition to this, Mosher also highlighted specific duties the captain might perform including to ensure a constant flow of information between the coach and team, to lead by example, to help the coach in the planning stages for the team and to conduct themselves in a professional manner before, during and after games. Dupuis et al. (2006) highlighted some common functions of ice hockey captains including being effective communicators, remaining positive and controlling their emotions. In professional football teams, having a good captain on the team has also been associated with better team member health and lower burnout (Fransen et al., 2020a). Although players and coaches have high expectations of their team captains, in practice, it seems that only few team captains can live up to these high standards (Fransen et al., 2019).

While many attempts have been made to describe the role of the captain, a strong evidence base has been lacking, particularly in terms of the demands of captaincy and the challenges faced. There is little consensus regarding the role of the captain, which can make it difficult to understand the context-specific demands of the role (Cotterill et al., 2019). One of the reasons for this is that the role can vary significantly from sport to sport, and across levels of performance (Cotterill and Cheetham, 2017). For example, in soccer, the captain is a formal leader on the pitch and a role model off it, but the way the team plays and major tactical decisions during the game are generally determined by the coach. In comparison, the sport of cricket adopts an enhanced role for its captains, with the captain making all the decisions on the pitch and also being part of the formal leadership structure off the pitch (i.e., captain, coach and director of cricket; Cotterill, 2014). This does not suggest that the role of the captain is less important in soccer compared to cricket but does highlight significant differences in the role. While captains are consistently suggested to be an important aspect of team performance, to date, there is currently limited research explicitly exploring the specific role of captain and its development in sport (Cotterill and Fransen, 2016).

#### Informal Athlete Leaders

In addition to athletes that are formally recognised as leaders, such as the team captain, some athletes also achieve their leadership status in an informal way, namely, through natural interactions with their teammates (Loughead et al., 2006). Regardless of their leadership status, both team captains and informal athlete leaders can occupy different leadership roles. Building on earlier athlete leader categorisations (Bales and Slater, 1955 Loughead et al., 2006), Fransen et al. (2014) advanced a 4-factor athlete leadership categorisation system, including four leadership roles that athlete leaders could undertake for the team: (a) *the task leader*, who gives teammates tactical advice and adjusts them when necessary; (b) *the motivational leader*, who encourages teammates to perform at their best; (c) *the social leader*, who develops a good team atmosphere; and (d) *the external leader*, who handles the communication with club management, media and sponsors. The study conducted by Fransen et al. (2014) emphasised the relevance of this leadership classification by demonstrating that an effective fulfilment of the four leadership roles by members of the team resulted in higher team confidence, stronger team identification and better team performance outcomes (e.g., ranking). Expanding upon the work of Fransen et al. (2014), Maechel et al. (2020) suggested an additional change-oriented leadership role, focused on promoting change and innovation and encouraging team learning.

The notion that leadership within teams is not just the preserve of team captains was suggested by Fransen et al. (2014). In a large cross-sectional study with 4,451 athletes and coaches across nine different sports, it was reported that almost half of the participants (44%) did not perceive their captain as the best leader on any of Fransen et al.’s four roles, neither on the field, nor off the field. These findings reported by Fransen et al. (2014) further highlight the importance of not restricting conceptualisations of leadership and associated leadership development to the team captain, but also more broadly to cultivate the leadership capacities of other leaders in the team, and potentially to all members of the team.

## Leadership Development

The ability to develop the leadership capabilities of individuals within a team can increase the likelihood that the leadership needs of a team are met (Cotterill, 2016). In this regard, enhanced leadership provision within a team has been linked to increases in team cohesion, athlete satisfaction, team identification, team confidence and the motivational climate within the team (Cotterill and Fransen, 2016). Building upon this point, in their study of professional sports teams, Fransen et al. (2017) reported that the team with the highest-quality athlete leadership on each of the four leadership roles excelled in all indicators of team effectiveness. More specifically, athletes in this team had a stronger shared sense of the team’s purpose, they were more highly committed to realising the team’s goals, and they had a greater confidence in their team’s abilities than athletes in the other teams. Moreover, this team demonstrated a higher task-involving and a lower ego-involving climate and excelled on all measures of performance. As a result, adopting a focus on enhancing leadership within their team is one way sports teams can seek to enhance team functioning and ultimately to positively influence both individual and team performance, while also having a positive impact upon health and wellbeing within the team (Fransen et al., 2019).

Leadership development has recently emerged as a scholarly discipline, separate and distinct from the more traditional approaches to studying leadership, such as the link between personality and leadership (Day et al., 2014). Across multiple domains, the required areas of knowledge, behaviours, skills and expertise have been identified as crucial building blocks for the development of effective leaders in sport. The concept of leadership development has been defined as involving expanding the collective capacity of team members to engage effectively in leadership roles and processes (McCauley et al., 1998).

Within the leadership development field, there had initially been some confusion between the different but related concepts of *leader* development and *leadership* development. According to Day et al. (2014), leader development focuses on developing the leadership ability of specific individual leaders (e.g., developing the leadership of a coach or a team captain), whereas leadership development focuses on a process of leadership development that involves multiple individuals (e.g., leaders and followers) designed to meet the leadership needs of the team and the context. While much historical research has focused primarily on leader development (e.g., cultivating traits and behaviours that characterise a good leader), leadership development has until recently received far less attention (Day, 2012). Day (2001) suggested that the optimal approach to the development of leadership in a specific context is to link leader development with leadership development such that the development of leadership ‘transcends but does not replace the development of individual leaders’ (p. 605).

In a sporting context, this distinction between leader development and leadership development is important because historically where ‘leadership development’ has taken place, there has been a tendency to focus on trying to develop the individual as a leader rather than to specifically seek to meet the context-specific leadership needs. Interestingly, it has also been suggested that many leadership training and development initiatives failed to produce effective leaders (Allio, 2006). This is mainly because these programmes have focused on promoting leadership literacy (e.g., by teaching leadership theory, concepts and principles) at the expense of developing leadership competencies. This finding emphasises that leadership needs to be learned to be effective, not just taught. Applied to a sporting context, this perspective on leadership development then advocates the importance of ‘on the job’ learning, with athletes getting the opportunity to develop their leadership abilities in practice. Illustrating the importance of this experiential learning in practice, Grandzol et al. (2010) found that serving as a team captain provided athletes with a rich opportunity to learn and practice their leadership skills. The authors suggested that effective programmes of leadership development should include the opportunity for future leaders to practice leading and applying leadership skills.

There has been a growing interest in athlete leadership development and the development of leadership among athletes within teams in the sport psychology literature over the last decade. Initially, there was a focus on the development of personal leadership skills in youth athletes through sport (e.g., Martinek and Hellison, 2009; Gould and Voelker, 2012; Gould et al., 2013), but more recently, there has begun to be an expansion in the studies exploring leadership development with adult athletes (Voight, 2012; Cotterill, 2016), the development of formal leaders, such as captains (e.g., Cotterill and Cheetham, 2017), and the implementation of shared leadership structures, which encompass the development of informal leaders as well (e.g., Mertens et al., 2020, 2021; Fransen et al., 2020d). We will first discuss the leadership programmes focusing on the team captain, after which we will elaborate on the leadership programmes targeting the broader team, which may encompass either all athletes on the team or selected leadership groups.

### Leadership Development With Team Captains

Several studies have examined how to develop the leadership ability of those members of the team who occupy formal (e.g., captain/vice-captain) positions within that team. The rationale behind this approach is that in many sports teams, the captain is perceived to fulfil an important leadership function. Not surprisingly, several published studies have reported the effectiveness of leadership programmes involving the development of team captains. At the youth level, Gould and Voelker (2010) created a 1 day workshop for high school captains on how to be an effective team captain. This programme involved learning about topics that included ‘What you need to know as a leader’ and ‘Handling common team problems’. At the end of the 1 day workshop, the participants were also given a guidebook, titled *Becoming an Effective Team Captain: Student-Athlete Guide*, focused on topics, such as the role of a team captain, effective communication, team motivation, team building and cohesion, handling tough team situations and recommendations from captains and coaches. One particular concern that needs to be highlighted when developing the leadership of youth sport team captains is the ongoing support and guidance provided to them by their coaches (Collins et al., 2009; Voelker et al., 2011). Part of the problem is that often the coaches are not sufficiently equipped or educated (in relation to leadership development) to develop the leadership skills and abilities of their athletes (Gould et al., 2013).

At the intercollegiate level, Voight (2012) oversaw a season-long athlete leadership development programme with two women’s volleyball teams directed to the leadership development of the team captains and assistants. The programme consisted of 15 stages (e.g., leadership assessment, leadership roles and responsibilities and captain platform) and was developed to help improve team communication and functioning, to assist the team daily and to foster the personal leadership development of the team’s formal leaders. To determine the programme’s effectiveness, two captains and two assistant captains were interviewed. Based on these interviews, the author concluded that the programme was effective in developing the leadership potential of these formal leaders as they indicated that the programme had a positive impact on their own personal leadership skills, enhanced their team’s cohesion and impacted both the team’s and individual teammates’ performances.

At the professional level, effective captaincy development programmes have been designed to reflect the specific requirements of this role within a specific sport. Once required knowledge, skills and expertise are identified, programmes that focus on prioritising those factors can be developed. For example, Cotterill (2016) developed a leadership development programme for elite (international) United Kingdom professional cricketers, building upon the key captaincy demands of this sport including: tactical decision-making, selection, player management, liaison with the coach and representing the team. This captaincy development was delivered through a focused ‘captaincy development’ group (a group of players elected as potential future captains) within the broader squad of players that focused on awareness of the self and others using the Myers-Briggs Type Indicator (MBTI) tool. This structured programme used a range of relevant guest speakers and offered all the players in the group the opportunity to get practical experience as a captain in practice games as part of the broader performance programme and to receive leadership performance debriefs from a sport psychologist and coaches. The programme in this case study focused on developing leadership at three distinct levels: (a) personal growth and leadership development; (b) leadership skill development; and (c) leader (captain) development. These three levels have been identified as crucial in helping to cultivate leaders at an international level of performance (Cotterill, 2016). Reflections on the programme by the participants suggest that a formal development programme can be beneficial in enhancing the leadership capabilities of elite captains. It is important to note that these findings are context-specific and while the approaches show promise, further research and exploration are required.

### Leadership Development of All Players Within the Team

Given the large variations in abilities needed to fulfil the leadership roles within sport teams, current thinking suggests that the most effective way to meet this diverse range of leadership needs within a team is to adopt a ‘shared’ approach to athlete leadership. Indeed, recent research adopting a shared approach to leadership has highlighted that leadership is often distributed within sport teams (Fransen et al., 2014, 2015; Leo et al., 2019). For instance, using a social network approach with four soccer teams, Duguay et al. (2019) found that every player was viewed by at least one other teammate as providing leadership to them. This finding underscores the importance of fostering the leadership development of all athletes beyond those who are captains.

This has been the approach taken by Duguay et al. (2016), who developed the leadership capabilities of all athletes, regardless of their leadership status, in two intercollegiate volleyball and basketball teams. The athlete leadership programme was grounded in Chelladurai’s (2007) Multidimensional Model of Leadership and Avolio’s (1999) Full Range Model of Leadership, where participants learned about numerous leadership behaviours and how these behaviours impacted the team’s dynamics. The leadership training was completed over the course of the regular season using four 1 h workshops. Each workshop consisted of a 3-step procedure, including: (a) a presentation of the leadership behaviours to be learned; (b) a demonstration of these leadership behaviours in action; and (c) the opportunity to practice these leadership behaviours. Throughout the workshops, the activities (e.g., role playing and case studies) highlighted how the leadership behaviours benefited the participants individually and how they enhanced the psychological factors grouped under the title team dynamics (cohesion, communication, motivational climate and satisfaction). The results from pre- and post-intervention indicated that the leadership programme enhanced eight of the 10 leadership behaviours (i.e., training and instruction, democratic behaviour, social support, positive feedback, appropriate role model, inspirational motivation, high-performance expectations and fostering acceptance of group goals and promoting teamwork). In addition, the findings concerning the team’s dynamics showed increases in athlete satisfaction and motivational climate, while maintaining levels of cohesion and communication over the course of the season.

Another approach to shared athlete leadership development of all athletes was advanced by Maechel et al. (2021) using a solution-focused approach. This approach assumes that for shared leadership to develop, teammates communicate with one another through the exchange of ideas, values and information. As a result, this approach also assumes that athletes can and have the resources to effect changes within their team with the assistance of a facilitator (e.g., sport psychology consultant). The authors delivered four separate workshops over the course of the season to three teams (while three other teams served as a control group). These four workshops were designed using the four athlete leadership meta-categories advanced by Maechel et al. (2020) that contained social, task, change and external-oriented forms of leadership. Using a mixed-methods approach, the quantitative data showed that all four meta-categories were significantly higher for the three teams in the intervention condition compared to the control condition. Further, the qualitative aspect of the study indicated that the intervention enhanced communication among team members, increased interpersonal relationships among teammates, whereby teammates got to know each other better, which in turn led to enhanced team cohesion, enhanced coach-team interactions with better communication and contributed to the processes of enhancing the shared nature of leadership within the team (e.g., transitioning from a few athletes leading the team to the whole team displaying varying forms of leadership).

### Leadership Development With Leadership Groups

#### The Importance of Leadership Groups

Athlete leadership groups are a designated group of athlete leaders from within the team who either provide shared leadership or support the decision making of a formal athlete leader (Cotterill et al., 2019). As the previous paragraphs outlined, shared leadership can encompass a range of leadership structures that vary in the extent and manner of sharedness, ranging from the sole focus on the team captain to involving all athletes within the team. Current perspectives have argued that neither one of these extremes is optimal. It is likely that not all team members will have the requisite skills and/or motivation to lead (Seibert et al., 2003). More importantly, if all team members assume leadership roles, then the task of coordinating their messages is considerable, and the difficulty of doing this increases the likelihood of confusion and miscommunication (Eys et al., 2007). As Gockel and Werth (2010, p. 179) observed as: ‘It might be good to share the burden of leading, but too many cooks might spoil the broth’. Conversely, minimal shared leadership structures, involving only two team members (e.g., the coach and team captain), do little to address problems associated with leadership role overload (Turner, 2002). Here, then, individuals could potentially have more roles than they have the time, energy or expertise to perform, creating role conflict that can put them under considerable strain (Fransen et al., 2014).

Taken together, the evidence would suggest that optimal leadership sharedness can be found somewhere between the minimal and maximal extremes. Consistent with this assumption, there is evidence that the relationship between the number of appointed leaders in a shared leadership structure and team outcomes is curvilinear (Eys et al., 2007 Gockel and Werth, 2010; Fransen et al., 2018; Leo et al., 2019). Specifically, an intermediate level of shared leadership is preferable to having either too few leaders or too many. In this regard, working with leadership groups addresses the need to steer a middle path by combining vertical and shared leadership in a way that distributes formal leadership responsibilities broadly—but not too broadly—within the team.

Also in professional sport, there is an increasing focus on the use of leadership groups and the adoption of formal leadership groups by coaches to meet the perceived leadership needs of the team (Haddad et al., 2021). In professional football in Australia, for example, coaches advocated the use of leadership groups as they believed that player ownership and autonomy regarding leadership had a positive impact upon performance, upon on and off field functioning, and ultimately upon the team’s culture (Haddad et al., 2021). Fransen et al. (2017) corroborated these assumptions and showed that the quality of those leadership teams within Australian professional football teams indeed predicted their effectiveness. More specifically, athletes in the team with the highest-quality leadership team had a stronger shared sense of the team’s purpose, they were more highly committed to realising the team’s goals and they had a greater confidence in their team’s abilities than athletes in the other teams. Moreover, this team demonstrated a higher task-involving and a lower ego-involving climate and excelled on all measures of performance. In line with this work, Mertens et al. (2021) showed that as teams that grew towards more shared leadership throughout a season they also experienced improvements in their functioning and performance.

#### Creating Leadership Groups

Realising that the quality of these leadership groups is a key predictor of the team’s effectiveness, an important step in creating these leadership groups is to identify the optimal leaders within the team. Here, it is suggested to develop clarity regarding different leadership roles in the leadership group (e.g., as task, motivational, social or external leaders; Fransen et al., 2020a). This role differentiation will also foster role clarity so that leaders can focus on the clearly defined responsibilities attached to their specific role. Previous evidence highlights this role clarification as one of the cornerstones of successful team development interventions (Shuffler et al., 2011) as it also cultivates greater role efficacy and enhanced role performance in sport teams (Bray and Brawley, 2002).

To identify the best leaders on the team on each of these roles, it is critical to look beyond the team captain, as often the informal athlete leaders are the real drivers of the team’s success (Fransen et al., 2020d). While coaches are often keen on appointing the leaders themselves, it seems that in most teams, coaches and athletes do not agree on who the best leaders are in their team, suggesting that the acceptance of athlete leaders who are chosen by the coach is likely to be insufficient to obtain effective leadership (Fransen et al., 2020d). To obtain the necessary insight in the leadership structure within the team on specific leadership roles, Shared Leadership Mapping can be used (Fransen et al., 2020b). This is a diagnostic tool that uses social network analysis to map all leadership perceptions in the team with the aim of identifying the best leaders on the team on each role (Fransen et al., 2020d).

After identifying the most suitable leaders in the team, it is also important to formally appoint them in their role (e.g., as task, motivational, social or external leaders). As this formal appointment is based on the perceptions of other athletes, athlete leaders will realise that their leadership is accepted and appreciated by their team. This support base will boost their motivation to fulfil their leadership role well and to take on their responsibility, especially in difficult times (Cotterill and Fransen, 2016; Fransen et al., 2020a). Therefore, one could argue that the appointment of these athlete leaders is already a first important step in the leadership development process.

#### Enhancing the Leadership Quality of the Leadership Group

Given the importance of the quality of these leadership groups, in the next stage, the leadership potential of the appointed athlete leaders should ideally be further developed. In recent years, an alternative approach to understanding effective leadership within sport teams has posited that athlete leaders are only effective to the extent that they are able to create and manage a shared social identity within their team (Haslam et al., 2020; Fransen et al., 2020a; Stevens et al., 2021). In other words, these leaders encourage their teammates to not only think, feel and behave as individuals (in terms of personal identity as ‘I’ and ‘me’), but also, and more importantly, as group members (in terms of a shared social identity as ‘we’ and ‘us’). This leadership quality in which the best athlete leaders distinguish themselves from others is also termed identity leadership.

A programme that specifically aims to build leaders’ identity leadership skills is the 5R Programme originally designed for formal leaders in organisational settings (Haslam et al., 2017). The 5R Shared Leadership Programme (or in short *5R^S^*) tailors the programme to the sport context and adds the benefits of implementing a structure of shared leadership (Fransen et al., 2020c). More specifically, 5R^S^ involves two steps. In a first step, Shared Leadership Mapping is used to identify the best task, motivational, social and external leaders in the team. After formally appointing these athlete leaders to this role, in a second step, these leaders guide their teams throughout five workshops (i.e., the 5R’s), in which they learn in an applied setting how to provide identity leadership. More specifically, the first Readying phase seeks to demonstrate why ‘we’ matter building commitment to the programme through informing the team members about the importance of group and social identity processes. In the *Reflecting* phase, leaders clarify people’s understanding of what the group stands for by guiding their team through the process of defining its core values and seeking to understand their shared social identity. In the next *Representing* and *Realising* phases, the leadership group then works together with their team to bring this social identity into practice. More specifically, the team identifies shared team goals that represent their core identity and develop strategies that help them in achieving these goals. After the team has had sufficient time to put their strategies into practice and attain their goals, the final *Reporting phase* involves assessing the progress towards the identified goals and evaluating the effectiveness of the adopted strategies. This programme adopts a team-centred approach, where workshops are delivered to the entire team and where the appointed athlete leaders are given additional responsibilities to learn practical skills relating to how to take the lead.

Qualitative data from two initial implementations with an organisational team (Belgian University administrator team) and a sport team (female volleyball team) revealed that participants positively evaluated the programme and showed the benefits of 5R^S^ for the team’s functioning (Fransen et al., 2020b). Further building on the qualitative insights from these case studies, some recent intervention work further supports the effectiveness of 5R^S^. In a first step, Slater and Barker (2018) adopted the core three Rs of the second phase of this programme (i.e., reflecting, representing and realising) in an intervention study with an elite disability football team. Instead of guiding the entire team throughout the programme, these researchers focused on delivering the programme to a senior leadership group consisting of three coaches and four senior athletes. Their longitudinal data showed that the core 3R’s had a positive effect on perceived identity leadership and athletes’ identification with their team, although these increases were only significant in the second year of the programme. Furthermore, qualitative data supported that the intervention helped in building connectedness within the team.

More recently, Mertens et al. (2020) conducted an experimental-control group intervention with eight national-level basketball teams. The results revealed that the 5R^S^ programme was successful in strengthening athlete leaders’ identity leadership skills that also served to increase team members’ identification with their team. Furthermore, in contrast to athletes in the comparison condition, athletes in the 5R^S^ condition were able to maintain their levels of intrinsic motivation and commitment to team goals, while also reporting improved wellbeing. In a follow-up study, Mertens et al. (2021) tested the effectiveness of 5R^S^ by conducting a wait-list controlled trial with a larger sample (i.e., 16 basketball teams). The authors reported that the 5R^S^ programme enhanced athlete leaders’ identity leadership skills, strengthened athletes’ identification with their team, enhanced the perceived social support available in the team, helped athletes to remain motivated and confident in their abilities and nurtured athletes’ health.

## Driving Forces Behind Shared Leadership Structures

### Coach-Led Leadership Development

Given that coaches play a vital role in the development of athlete leadership, this section focuses on the role of the coach in enhancing athlete leadership and athlete leadership development. Duguay et al. (2020) examined how coaches nurtured and developed athlete leadership within their teams. Through interviews with 15 intercollegiate coaches, four overarching themes were developed relating to how coaches facilitated the development of athlete leaders. The first theme revolved around the significance of empowering their athletes. The coaches noted that to develop athlete leadership, it was critical to be athlete-centred and this required the athletes to be involved in some of the decision-making around team matters, encouraging the athletes to take initiatives related to team activities, such as team building and community events. The second theme concerned how coaches utilised the concept of team leadership. The coaches expressed the belief that the leadership of the team was too large a responsibility for just one athlete. Instead, the coaches preferred the use of leadership groups. The size and composition of these leadership groups were not universal and were dependent on factors, such as the number of veteran athletes on the team and the maturity of the athletes. In some cases, coaches had leadership teams of 5–6 players composed of 1–2 captains along with future/promising athlete leaders. In other cases, coaches rotated the team’s captaincy and designated athletes for different leadership roles (e.g., academic captains and weight room captains). The third way in which coaches supported the development of athlete leadership was through the creation of a positive team culture for leadership to flourish. The coaches created a team environment that eliminated status differences between athletes (e.g., rookies vs. returning players), making sure that all athletes had a voice through the facilitation of open communication, developing trust and having team-building activities. Lastly, coaches discussed the importance of intentionally developing the leadership capabilities of their athletes. The coaches emphasised the importance of having experiential learning opportunities including team discussions on the importance of displaying effective leadership, providing books to their athletes on leadership, having leadership workshops and modelling effective leadership behaviours among the coaching staff. Some coaches have also adopted a mentoring approach to the development of athlete leadership and athlete leaders (e.g., Mead and Gilson, 2017). Coaches have previously highlighted that a lack of clarity regarding the role of the athlete leader and the skills they need to be successful has hampered coach-led athlete leader development (Cotterill et al., 2019).

### Mentorship Approaches

A relatively recent advance in the leadership development literature relating to sport is the application of mentorship approaches (Mead and Gilson, 2017) to enhance the leadership ability of individual leaders. In this approach, a more experienced leader (e.g., the team’s coach or a senior athlete) trains a protégé by consistently interacting and sharing ideas (Day, 2001). The effectiveness of this approach relies heavily on how positive the relationship is between the mentor and the protégé (Riggio, 2013). In a study of American high school basketball, Mead and Gilson (2017) explored the impact of coach mentoring on athlete leadership development. The study itself provided a rich and detailed description of the coach’s approach to mentoring, and his successes and failures. Specifically, the coach sought to allow formal leaders to use their personal voice, distribute and delegate leadership tasks to these leaders, offer reminders of important leadership concepts and set an effective example as coach. The captains in this study were also encouraged to reflect on their own leadership development, an approach that has been suggested to be an important part of the leader development process (Grandzol et al., 2010). The coaches are in a great position to role model desired prosocial behaviours and to create an environmental culture in which cooperation and skill development are emphasised to further foster personal growth and prosocial behaviour (Kavussanu et al., 2006).

In addition to coaches, athletes mentoring one another has been used to foster leadership development. Hoffmann et al. (2017) defined peer athlete mentoring as a dynamic process where a more experienced and knowledgeable athlete, serves as a trusted role model to another athlete, referred to as the mentee, assisting the mentee in achieving their goals along with supporting their personal growth and development. While it is widely recognised that many athletes benefit from being mentored, it should be highlighted that many athletes never get to experience these benefits. It has been reported that nearly 40% of Canadian intercollegiate athletes never considered another athlete as a peer mentor (Hoffmann and Loughead, 2016), and 25% of a sample of Canadian National team and intercollegiate athletes have never been peer mentored (Hoffmann and Loughead, 2019).

Consequently, Hoffmann (2019) suggested several strategies for those interested nurturing peer athlete mentoring relationships. In general, two broad strategies can be utilised, whereby the first allows for mentoring relationships to develop informally. Informal mentoring relationships are preferable due to their natural occurrence between mentor and mentee (Hoffmann, 2019). Informal mentoring stems from a process of mutual discovery where mentors and mentees identify with one another. The second is to formalise peer mentoring relationships among athletes whereby athletes are assigned to their mentor using one of three strategies: (a) where the practitioner assigns the mentee to a mentor, (b) a choice based approach where the mentors and mentees mutually agree to engage in a mentoring relationship, and (c) an assessment-based approach where the compatibility between mentor and mentee is derived through some type of assessment tool (e.g., personality questionnaire, such as the NEO Five-Factor Inventory; Costa and McCrae, 1992).

The enhancement of leadership within sports teams has been positively linked to team functioning and team-related outcomes (Cotterill, 2016), and as a result is an important aspect of team functioning. The increased focus on leadership development in recent years has provided the opportunity to positively impact on athlete leadership in an evidence-informed way. Seeking to enhance leadership development at different levels within the team (i.e., formal leader, informal leader or leadership group level) provides different ways in which team leadership provision can be enhanced.

## Future Directions for Research and Practice

While there has been an increasing focus on leadership development in sport in recent years, there are still a number of important questions that remain unanswered and areas that need further exploration relating to both research and applied practice. Of particular importance is understanding how best to set about seeking to apply both leader and leadership development knowledge to enhance the delivery of leadership development programmes. Historically, much of the leadership development activities and programmes outlined in this article have been delivered in a traditional face-to-face format. Recent developments though have seen the development of online leadership develop programmes, allowing for broader and more flexible engagement. For example, Pierce et al. (2018) developed an online course for high school captains that could be accessed free of charge. The course was composed of video narration, personal leadership vignettes and interactive web-based activities, taking around 2 h to complete. This type of approach could be replicated in other domains with different populations.

Future research should also seek to explore both the impact and effectiveness of athlete leadership development programmes that seek to utilise different modes of delivery (e.g., online vs. face-to-face and synchronous vs. asynchronous). The majority of athlete leadership research to date has understandably focused on sport teams. These are, though, not the only domains in which athletes are required to demonstrate leadership abilities. Future research should also explore the leadership needs and approaches to leadership development in individual sports as well. This is an area that has received very little attention in the published literature to date.

Across the athlete leadership literature, the leadership needs of different sports have been explored, though the range of different sports is still relatively narrow. A better understanding of the leadership needs within specific sports will enable better focused leadership programmes and activities to be developed within those sports. In some sports (such as cricket), the formal leader (captain) is a crucial aspect of the game, but for some other sports, the need for a specified formal leader is far less clear. As a result, the leadership and leader development needs of different sports may vary greatly.

For sports in which the position of a captain is required, understanding how to select the best candidate for this formal position without suppressing the leadership potential in the rest of the team is key. Previous research has highlighted a particular disconnect between the demands of the captaincy role and the process of selection and appointment (Cotterill et al., 2019). Also, Fransen et al. (2019) showed a clear discrepancy between what players and coaches expect from their captain and the criteria used to select team captains. Therefore, having a good insight in the most effective internal leadership structure for a team is important before making this decision.

Even though a formal captain position might be required, it is still important to invest in broader leadership development in the team to fully harness the leadership potential of the team. There is evidence to imply that the relationship between the centralisation of the leadership networks and the outcomes is curvilinear, suggesting that the most effective leadership structures are those with a limited number of leaders, neither too few, nor too many (Eys et al., 2007 Gockel and Werth, 2010; Fransen et al., 2018; Leo et al., 2019). An outcome that implies that the development of leadership groups might well be the most effective way to meet the leadership needs of a team. Though it is important to recognise there should still be a focus of the development of individual leadership skills, particularly at a junior/developmental level (e.g., youth or academy level). In youth sport teams, having rotating leadership roles allows a greater range of athletes to get the chance to develop their leadership skills. As the area of athlete leadership has developed, it would also be useful to take stock of developments to date, potentially through the adoption of a meta-analysis and/or systematic review to best understand current knowledge and crucially gaps in our current understanding and approaches to practice. There are also significant differences in the size and scope of much of the preceding research cited. Some studies focus on brief interventions (i.e., single workshops) while a limited number has focused on a much longer timescale (i.e., over 6 months). There are unanswered questions relating to the development of athlete leadership and athlete leaders over time and the potential to explore the transitions of athlete leaders from one team to another. It is interesting to see in recent years an expansion of the methodological approaches adopted in athlete leadership research, such as social network analysis. In addition, researchers should also explore the application of different leadership approaches in other research fields that have focused on enhancing group or team leadership, with a view to generate an enhanced evidence base to underpin intervention approaches in sport.

## Conclusion

The last 10 years have seen a small but expanding literature that has sought to build upon the broader athlete leadership body of knowledge to better understand and report approaches to athlete leadership development. There is though still a long way to go, further clarity is required regarding the knowledge, skills and expertise required to undertake the athlete leadership roles in sport, and crucially to better understand how the development of current and future athlete leaders can be maximised.

## Author Contributions

SC, TL, and KF all contributed to the initial scoping of the review and were involved in the continued revision, extension and redrafting of the manuscript. SC took the lead in developing the initial content with TL and KF further expanding and extending this initial content. All authors contributed to the article and approved the submitted version.

## Conflict of Interest

The authors declare that the research was conducted in the absence of any commercial or financial relationships that could be construed as a potential conflict of interest.

## Publisher’s Note

All claims expressed in this article are solely those of the authors and do not necessarily represent those of their affiliated organizations, or those of the publisher, the editors and the reviewers. Any product that may be evaluated in this article, or claim that may be made by its manufacturer, is not guaranteed or endorsed by the publisher.
